# New Segmented Poly(Thiourethane-Urethane)s Based on Poly(ε-Caprolactone)Diol Soft Segment: Synthesis and Characterization

**DOI:** 10.3390/ma15144940

**Published:** 2022-07-15

**Authors:** Andrzej Puszka, Janusz W. Sikora

**Affiliations:** 1 Department of Polymer Chemistry, Faculty of Chemistry, Institute of Chemical Sciences, Maria Curie-Skłodowska University in Lublin, Gliniana 33, 20-614 Lublin, Poland; 2 Department of Technology and Polymer Processing, Faculty of Mechanical Engineering, Lublin University of Technology, Nadbystrzycka 36, 20-618 Lublin, Poland; janusz.sikora@pollub.pl

**Keywords:** transparent polyurethanes, surface free energy, mechanical properties, differential scanning calorimetry, thermoplastic polyurethanes, optical properties, poly(ε-caprolactone)diol

## Abstract

New segmented poly(thiourethane-urethane)s (SPTURs) were synthesized by the reaction of 1,1′-methanediylbis (4-isocyanatocyclohexane) (*Desmodur W*^®^, HMDI) and poly(ε-caprolactone)diol (PCL) and (methanediyldibenzene-4,1-diyl)dimethanethiol as nonconventional polymer chain extender. FTIR spectroscopy was used for the structural analysis of obtained polymers. The molecular weight distribution was examined by GPC chromatography. Based on the measured contact angles, free surface energy parameters were calculated. Thermal properties of polymers were examined by DSC and TGA, while viscoelastic properties were measured by DMTA. The tensile, adhesive and optical properties were also investigated for the obtained polymers. It was shown that SPTURs were transparent or partially transparent solids with high molar masses up to 84,300 Da. These polymers showed a good resistance to hydrolysis during incubation in Optylite^®^ physiological saline over 8 weeks. Obtained polymers possessed a tensile strength of up to 43.26 MPa, hardness of up to 96.25/59.00 Sh A/D and adhesion to copper of 14.66 MPa. The surface properties of the obtained polymers show that all obtained SPTURs were hydrophilic (CAs for water between 64.07° and 73.12°) with calculated SFE up to 46.69 mN/m.

## 1. Introduction

Due to the variety of their applications, polyurethanes play an important role in the global production of plastics [1,2,3,4,5,6]. They can be thermoplastic or thermoset. Thermoplastic polyurethanes (TPUs) are typically produced by reacting diisocyanates with both oligomeric and short-chain diols to form (A-B)_n_-type block copolymers [1,2,3,4]. One block of the polymer chain is referred to as the soft segment (SS), while the other block is referred to as the hard segment (HS) [3,4,5,7]. The soft segments give polyurethanes softness, flexibility, long elongation at break and low temperature resistance, while the hard segments have a particular influence on the modulus of elasticity, hardness and tear strength. Generally, the soft and hard segments undergo microphase separation due to thermodynamic incompatibility resulting in materials with excellent mechanical properties [2,4,7,8]. In conventional TPUs hard segments are built from short-chain diols, mostly butane-1,4-diol (BD) and diisocyanates, such as 4,4′-diphenylmethane diisocyanate (MDI), 4,4′-dicyclohexylmethane diisocyanate (HMDI) [9,10] and hexane-1,6-diyl diisocyanate [9]. TPUs derived from MDI show the best mechanical properties; however, due to the toxic properties of aromatic diisocyanates, polyurethane materials based on aliphatic diisocyanates are preferred for biomedical applications [9,11].

Oligoester, oligoether or oligocarbonate diols are the most commonly used oligomeric diols for the synthesis of conventional thermoplastic polyurethanes [3,5,6,12,13]. The TPUs with the polyether soft segments are characterized by better low-temperature properties and hydrolytic resistance than the TPUs with the polyester soft segments but they are susceptible to oxidation. Improved oxidative resistance as well as hydrolytic stability is shown by the TPUs with polycarbonate soft segments. Because of their combination of excellent biostability and biocompatibility as well as high tensile strength and modulus they are preferred as biopolymers for long-term implantation [12,13]. In turn, polyurethane materials, for the synthesis of which poly(ε-caprolactone) soft segment was used, have received special interest in recent years [14,15,16,17,18]. Due to their good biocompatibility and biodegradable properties, PCL-based polyurethanes have been used, among others, in biomedical materials [8,9,14,15].

This work is part of ongoing research on the modification of typical TPUs with unconventional chain extenders containing sulfur atoms [19,20,21,22,23,24,25]. The presence of sulfur atoms in polymer structure, depending on the kind of functional group, can improve some important properties, e.g., thermal and chemical resistance and optical properties (refractive index).

In this study, various segmented poly(thiourethane-urethane)s (SPTURs) were synthesized by using cycloaliphatic 1,1′-methanediylbis(4-isocyanatocyclohexane) (*Desmodur W*^®^, HMDI) and sulfur-containing chain extender (methanediyldibenzene-4,1-diyl)dimethanethiol (dithiol) forming hard segments in the obtained polymers. The soft segments were composed of PCL. By applying the one-step melt method, we obtained SPTURs with HS contents of 30, 40, 50, and 60 wt%. The effects of composition on the properties of the resultant SPTURs were examined. For the synthesized polymers, structure by Fourier transform infrared spectroscopy (FTIR), the physicochemical (reduced viscosities, gel permeation chromatography (GPC), contact angles (CAs) and surface free energy (SFE), optical (refractive index, transparency and colour), thermal (differential scanning calorimetry (DSC) and thermogravimetric analysis (TGA)), thermomechanical (dynamic thermal mechanical analysis (DMTA)), mechanical (hardness and tensile test) and adhesive properties were investigated.

## 2. Materials and Methods

### 2.1. Materials

Dithiol (*T_m_* = 85 °C after recrystallization from ethanol) was obtained from bis (chloromethyl)-compound through the thiouronic salt [19,26]. HMDI (*Desmodur W*^®^) was kindly supplied by Covestro (Leverkusen, Germany). PCL of ${\bar{M}}_{n}$ = 2000 g/mol was purchased from Aldrich (St. Louis, MO, USA). Before being used, PCL was heated at 90 °C in vacuo for 10 h. Dibutyltin dilaurate (DBTDL) from Merck-Schuchardt (Hohenbrunn, Germany) was used as received. The analytical reagents were as follows: 1,1,2,2-tetrachloroethane (TChE), diiodomethane and tetrahydrofuran (THF) (Aldrich, St. Louis, MO, USA), 1–bromonaphthalene (POCh S.A., Gliwice, Poland), redistilled water (Millipore, UMCS, Lublin, Poland) and Optylite^®^ physiological saline (KabiPac, Kutno, Poland) were used in purchased form.

### 2.2. Measurements Methods

#### 2.2.1. Fourier Transform Infrared Spectroscopy (FTIR)

The FTIR spectra were developed by applying attenuated total (internal) reflection (ATR/FTIR) with the use of a Bruker TENSOR 27 spectrophotometer (Ettlingen, Germany), equipped with a PIKE measuring cell which features crystalline diamond embedded in zinc selenide. The FTIR spectra were recorded within the range of 4000 to 600 cm^−1^, with 128 scans per sample, at a resolution of 2 cm^−1^ in the absorption mode. The SPTURs were in the form of the compression-molded 1 mm thick sheet.

#### 2.2.2. Physico-Chemical Characterization

- Gel permeation chromatography (GPC):
  The number (${\bar{M}}_{n}$) and mass (${\bar{M}}_{w}$) average molar mass (Daltons (Da)), and the molar-mass dispersity (*Ð_M_*, *Ð_M_* = ${\bar{M}}_{w}$/${\bar{M}}_{n}$) [27] of the segmented polyurethanes were determined by gel permeation chromatography (GPC) performed on a Viscotek GPCMax (Westborough, MA, USA) equipped with Triple Detector Array TDA305. The eluent was tetrahydrofuran (THF), the flow rate was 1 mL/min, the operation temperature was set to be 35 °C and the molar mass was calibrated with polystyrene standards;
- Reduced viscosities:
  Reduced viscosities (*η*_red_, dL/g) of 0.5% polymer solution in (TChE) were measured in an Ubbelohde viscometer (Gliwice, Poland) at 25 °C;
- Contact angles (CAs) and Surface Free Energy (SFE):
  Contact angles (CAs) of SPTURs were measured at 20 °C with a contact angle goniometer (KRÜSS GmbH, Hamburg, Germany) with water and diiodomethane droplets. The volume of droplets was 2 μL. Each sample was analysed five times, and the average value of the contact angle was designated. For calculation of the surface free energy, according to the method of Owens, Wendt, Rabel and Kaelble [9], the Krüss ADVANCE (KRÜSS GmbH, Hamburg, Germany) software was used. The sessile drop orientation and the ellipse fitting method were used for the data analysis;
- Hydrolytic resistance:
  SPTURs samples weighing about 1 g were placed in a Optylite^®^ saline solution with the following concentrations of ions (mmol/dm^3^): 141 Na^+^, 34 CH_3_COO^−^, 3 C_6_H_5_O_7_^3−^, 2 Ca^2+^, 5 K^+^, 1 Mg^2+^, 109 Cl^−^ at 37 °C for a period of 8 weeks of immersion. After each week, the samples were taken from the solution, washed in distillated water and dried in vacuum at 60 °C and changes in their mass (%) were determined.

#### 2.2.3. Thermal and Thermomechanical Properties

- Differential Scanning Calorimetry (DSC):
  DSC thermograms were obtained using Netzsch 204 F1 Phoenix calorimeter (Günzbung, Germany), in accordance with standard ISO 11357-1:2016 [28]. The sample of 10.0 ± 0.05 mg was weighed and was first cooled and isotherm for 3 min at −100 °C and then heated up to a maximum temperature of 200 °C, next cooled to −100 °C and then heated to 200 °C. The scans were performed at the heating/cooling rate of 10 °C /min under argon atmosphere (gas flow = 30 cm^3^/min). All DSC measurements were taken in aluminum pans with a pierced lid (a mass of 40 ± 1 mg). As a reference, an empty aluminum crucible was applied. The reported transitions were taken from first and second heating scans. Glass-transition temperatures (*T_g_*s) for the polymer samples were taken as the inflection point on the curves of the heat-capacity changes. Melting temperatures (*T_m_*s) were read at endothermic-peak maxima;
- Thermogravimetric Analysis (TGA):
  TGA was performed with a Netzsch STA 449 F1 Jupiter thermal analyzer (Selb, Germany) in the range 30–800 °C in helium and in air (gas flow = 20 cm^3^/min) at the heating rate of 10 °C/min. Sample masses of about 10 mg were used. All measurements were taken in Al_2_O_3_ crucibles (with mass about 160 mg) and as a reference empty Al_2_O_3_ crucible was employed;
- Dynamic Mechanical Thermal Analysis (DMTA):
  DMTA of SPTURs was performed in tensile mode using DMA Q800 Analyzer TA Instruments (New Castle, DE, USA). Calibration was performed as per the manufacturer’s recommendations included in Advantage Software, version 5.5.24 (TA Instruments, New Castle, DE, USA). The experiments were carried out on rectangular samples of dimensions close to 1 mm thick, 5 mm wide and 30 mm long. Experimental conditions employed were frequency of 1 Hz and static stain 0.05% with the scanning temperature range from −100 °C to 150 °C in the air conditions and a temperature ramp of 3 °C/min. The samples were cut from the pressed sheets. The variations of storage modulus (E′), loss modulus (E″) and tangent delta (tan δ) versus temperature were determined.

#### 2.2.4. Mechanical Properties

Tensile testing was performed on a Zwick/Roell Z010 tensile-testing machine (Ulm, Germany) according to International Standard ISO 527-2:2012 [29] at the speed of 100 mm/min at 23 °C; the tensile test pieces 1 mm thick and 6 mm wide (for the section measured) were cut from the pressed sheet. Press molding was carried out with a Carver hydraulic press (USA) at 100–120 °C under a 10–30 MPa pressure. The hardness of the SPTURs was measured by the Shore A/D method on a Zwick 7206/H04 hardness tester (Ulm, Germany). The readings were taken after 15 s at the temperature of 23 °C [30].

The single lap-shear strength of the polymers to copper plate, 100 mm × 25 mm × 1.5 mm, was measured in accordance with Polish Standard PN-EN 1465:2009 [31] by using a Zwick/Roell Z010 (Ulm, Germany). The adhesive joint, 12.5 mm × 25 mm × 0.2 mm, was prepared by pressing the polymer between the ends of two copper plates (prepared according to PN-EN-13887:2005 [32]) and then leaving them under a pressure of 30 MPa to cooling to room temperature. Next, plates were fixed by tensile-testing machine clips and underwent tensile testing at speed of 2 mm/min at 23 °C.

#### 2.2.5. Optical Properties

- Refractive index (RI):
  RI was measured at 23 °C by using Conbest Abbe’s Refractometer Type 325 (Krakow, Poland) instrument according to Method A of European Standard EN ISO 489:2022 [33]. 1–Bromonaphthalene was applied between the sample and the prism shield;
- Transmittance:
  The ultraviolet–visible (UV/vis) spectra of the compression-molded sheets of the SPTURs were obtained by a UV-2550 (Shimadzu, Kyoto, Japan) UV spectrophotometer in the range of 200–900 nm and at a scanning rate of 200 nm/min;
- Colour:
  The colour measurement of samples was performed according to ASTM E308 [34], for which an X-Rite Ci4200 spectrophotometer was used. The colour is described in the CIELab system, where it is specified in L*, a* and b* space. Parameter a* describes the colour from green (negative values) to red (positive values); parameter b* is the colour from blue (negative values) to yellow (positive values) and parameter L* is the luminance—the brightness, representing the grey scale from black to white (value 0 corresponds to black and 100 to white).

### 2.3. Polymer Synthesis

**Figure 1:**
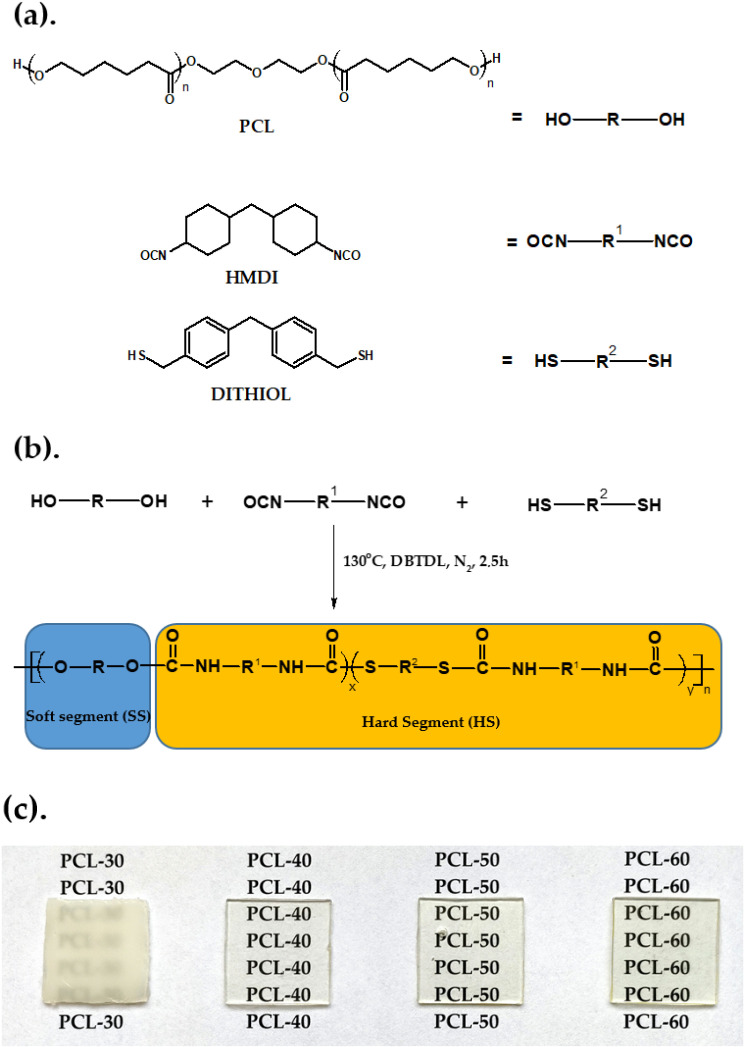
(**a**) Chemical structures of the reactants, (**b**) schematic representation of the synthesis route of polymers, (**c**) images of the synthesized polymers.

SPTURs with hard-segment contents of 30, 40, 50 and 60 wt% were prepared by a one-step melt polymerization from dithiol, HMDI and PCL at the NCO/(OH + SH) molar ratio of 1.07. The general procedure for the synthesis of SPTURs by this method was as follows. Oligomer diol and dithiol (0.01 mol together) and HMDI (0.0107 mol) were heated with stirring under dry nitrogen to 90 °C in an oil bath. A catalytic amount of DBTDL (about 0.03 g) was added to the formed clear melt and polymerization rapidly began at vigorous stirring. The reaction temperature was slowly elevated to 130 °C and the formed rubber-like product was additionally heated at this temperature for 2 h. Schematic representation of the polymer synthesis was given in Figure 1. Designations and composition were used to synthesize the SPTURs are given in Table 1. The polymers were designated as X-Y, where X is the abbreviation of soft segment and Y represents the hard-segment content.

## 3. Results and Discussion

The polymers obtained were transparent or partially transparent solids (see Figure 1c). All these polymers dissolved at room temperature in THF, TChE, chloroform, *N*,*N*-dimethylformamide and *N*,*N*-dimethylacetamide but they were insoluble in *N*-methyl-2-pyrrolidone. All polymers swelled in dimethyl sulfoxide.

### 3.1. FTIR

**Figure 2:**
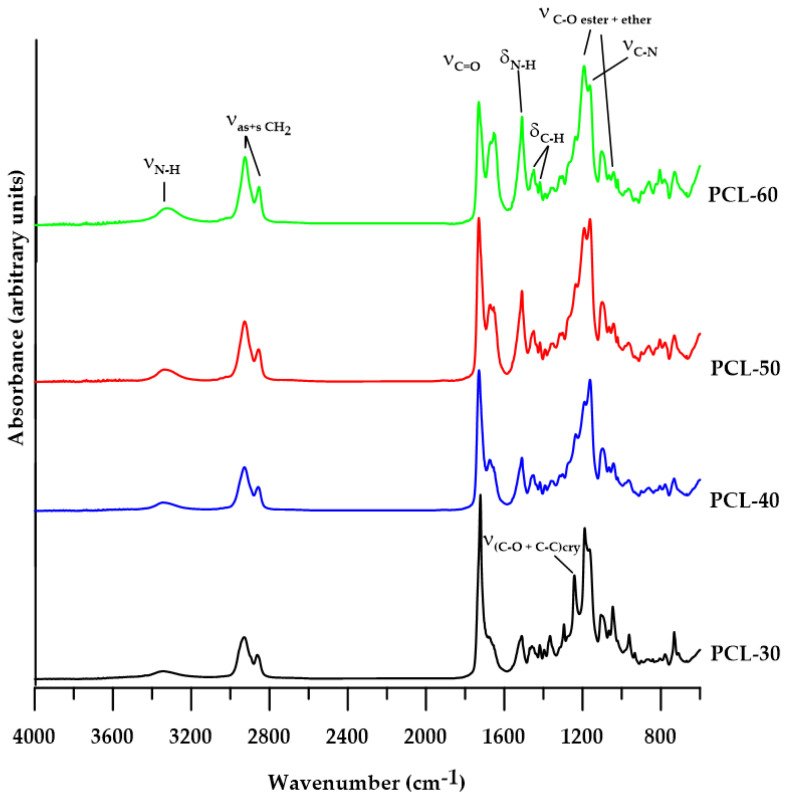
FTIR spectra of synthesized polymers.

The chemical structures of all the polymers were confirmed by FTIR spectroscopy. The characteristic bands of different groups present in SPTURs are visible in the FTIR spectra (see Figure 2).

The analysis of the FTIR spectra of the obtained polymers revealed the bands at about 3310 cm^−1^ (attributed to N-H stretching vibrations), 1510 cm^−1^ (attributed to N-H bending vibrations) and 1190 cm^−1^ (attributed to C-N stretching vibrations) of the urethane and thiourethane groups. The visible broad absorption bands in the range of 1730–1650 cm^−1^ are responsible for the vibrations of the C=O group of urethane, thiourethane and ester bonds. When analyzing this range, the individual bands can be assigned as follows: 1730 cm^−1^ (nonbonded C=O stretching of the ester group from PCL), 1720 and 1700 cm^−1^ (nonbonded and H-bonded C=O urethane group, and H-bonded C=O ester group), 1676 cm^−1^ (nonbonded C=O thiourethane) and 1654 cm^−1^ (H-bonded C=O thiourethane group). The presence of an ester bond in the obtained polymers is also confirmed by the absorption bands at approx. 1250 cm^−1^ and 970 cm^−1^ (asymmetric and symmetric C-O stretching, respectively) and about 790 cm^−1^ (C-O bending). In addition, PCL-based SPTURs exhibit absorption bands are characteristic of C-O stretching vibrations of the ether group at around 1065 cm^−1^. The FTIR spectra of both series of polymers also show the C-H stretching vibrations of CH_2_ groups at approx. 2933 cm^−1^ and 2858 cm^−1^ (asymmetric and symmetric, respectively) and bending vibrations C-H at about 1455 cm^−1^ (for cyclohexane ring) and at around 1370 cm^−1^ (for the vibration of the CH_2_ group of the aliphatic chain).

The FTIR spectrum of the PCL-30 polymer shows the absorption band at 1294 cm^−1^, which is responsible for the C-O and C-C stretching vibrations of the crystalline phase [35]. The disappearance of the intensity of this band for polymers with a higher HS content indicates that their crystallinity is also decreasing.

The absence of an –NCO band at about 2260 cm^−1^ shows that all –NCO groups were converted to urethane and thiourethane groups.

The changes in the intensity of the individual bands are closely related to the changes in the chemical composition of different polymers.

### 3.2. Physico-Chemical Characterization

#### 3.2.1. Reduced Viscosities and GPC

Reduced viscosities values of the obtained polymers (given in Table 2) ranged from 1.81 to 2.43 and in each series decreased with the increasing of the SS content in the sample. The obtained SPTURs were materials, which exhibits high molar masses ($\bar{M_{n}}$ in the range of 31,800–84,300 Da and $\bar{M_{w}}$ in the range of 57,200–130,000 Da) and relatively low dispersities (*Ð_M_* ranged from 1.30 to 1.80). The low molar mass dispersities of the obtained polymers results from the relatively long time of mixing the contents of the reaction flasks during the syntheses. This is due to the fact that at 135 °C all polymers were in a plasticized state. This indicates the high homogeneity of the polymers obtained.

#### 3.2.2. CAs and SFE

**Figure 3:**
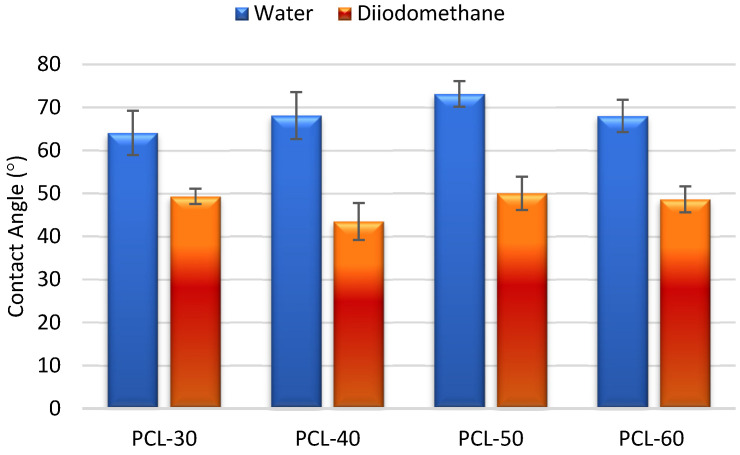
Quantifying the contact angles of SPTURs.

**Figure 4:**
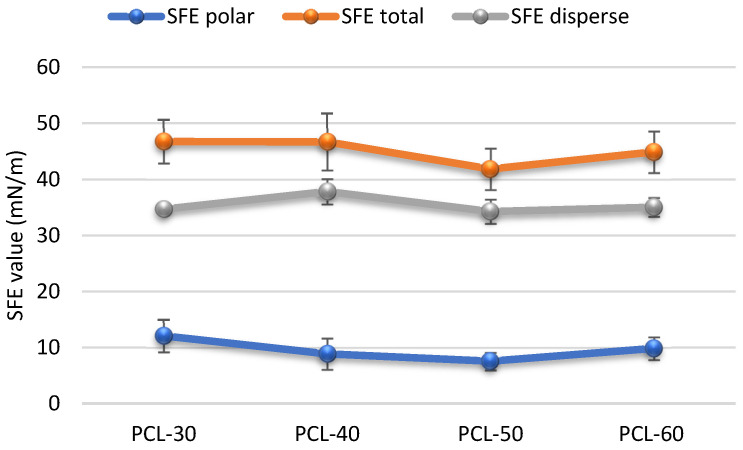
SFE values of synthesized polymers.

The surface properties of the obtained polymers were determined on the basis of the CA and SFE values, and the obtained results are shown in Figure 3 and Figure 4.

For material applications, biomaterial chemical characters and surface properties are of crucial importance. Both hydrophobic and hydrophilic properties may be preferred depending on the type of used biomaterial. The contact angle and SFE values are important to determine these properties. For polyurethanes materials, the SFE values are above 50 mN/m, so they are polar.

It is commonly accepted that hydrophilic surfaces have CA values up to about 30° and hydrophobic surfaces above 90°. As can be seen from Figure 3, all obtained SPTURs were hydrophilic (CAs for water between 64.07° and 73.12°). When analyzing the impact of changing the HS content in polymers, it can be seen that CA values for water increased, while SFE values decreased (except for PCL-60). In general, increasing the amount of HS in the polymer (and thus reducing the amount of polar ester and ether groups contained in PCL) results in an increase in the hydrophobicity of the materials obtained (increasing CAs values and decreasing SFE values).

#### 3.2.3. Hydrolytic Resistance

**Figure 5:**
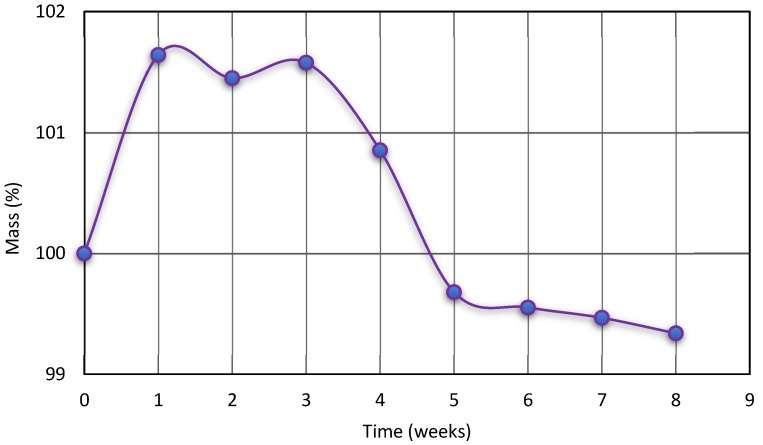
Diagram of sample mass change during Optylite^®^ liquid incubation.

The hydrolytic resistance of polyurethane material in the environment of the human body is extremely important for biomedical applications. To pre-assess this property, the SPTURs with 50 wt% was incubated in Opylite^®^ saline solution for 8 weeks. The observed changes in mass of the test sample are presented in Figure 5.

As can be seen in the Figure 5, in the initial stage of incubation, the physiological liquid was absorbed by the polymer chain (in this stage the polymer was swollen). After appropriate saturation with the solution (and loosening of the polymer chain), the hydrolysis of weakly resistant ester bonds took place, which shows the weight loss of the tested sample visible on the curve.

It is noteworthy that after 8 weeks, the mass of the samples decreased by about 0.7%. Literature reports suggest significant changes in the hydrolytic stability of polyurethanes only in the longer incubation period [36,37,38].

### 3.3. Thermal and Thermomechanical Properties

#### 3.3.1. DSC

**Figure 6:**
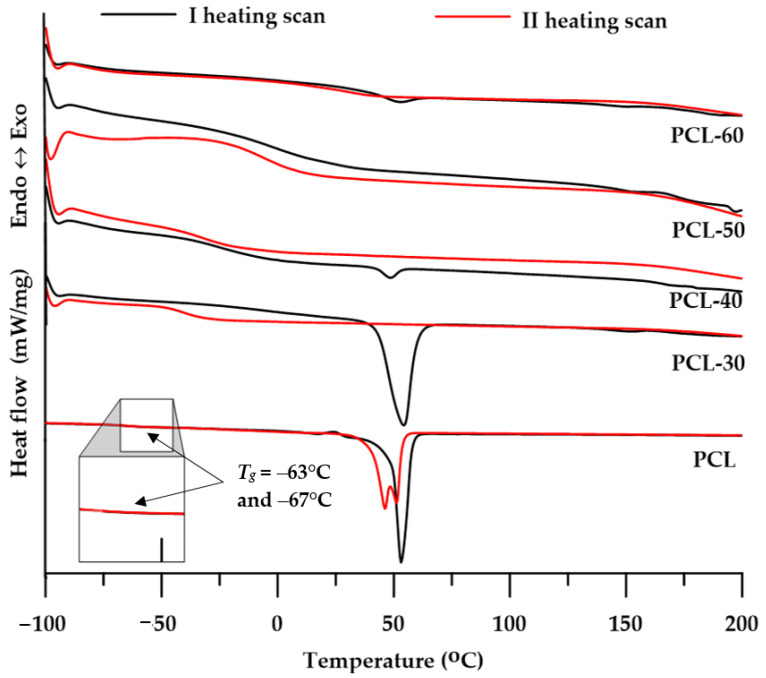
DSC curves of the synthesized polymers.

The changes in physical transformation of the obtained SPTURs were determined by DSC analysis. In order to better interpret the obtained results, the compounds that constituted SS in the obtained polymers were also tested. The numerical data of the analyzes are presented in Table 3, while the shapes of the DSC curves are presented in Figure 6.

The determined *T_g_* values of the obtained SPTURs were in the range of −30–42 °C in the first heating cycle and −39–13 °C in the second heating cycle. These values increased with the increase in the content of the HS in the polymers, with a significant increase being observed for polymers of 50 wt% of hard segments. On the basis of the differences in *T_g_* values of polymers and pure soft segments (PCL: −67 °C), it can be concluded that with the increase in the content of the hard segment in SPTURs, their microphase separation degree decreased. Polymers with a 60 wt% of the hard segment showed *T_g_* values slightly higher than room temperature, therefore they should be located on the border of elastomers and plastomers. More precise viscoelastic properties of the obtained materials were determined by the DMTA method, and the description of the obtained results is presented in the further part of the work.

On the DSC curves of all polymers (see Figure 6) from the 1st heating cycle, one or two endothermic peaks with *T_m_* values in the range of 49–192 °C were observed. The peaks with the lowest *T_m_* value in the range of 49–54 °C and Δ*H* values in the range of 0.3–50 J/g may be responsible for the melting of the soft segment. In turn, small endothermic peaks with *T_m_* values ranging from 151 to 192 °C should be attributed to the melting of more or less ordered hard segments [19].

The low Δ*H* values determined from the 1st heating cycle and the absence of endothermic peaks in the 2nd heating cycle curves indicate a slight tendency of the obtained SPTURs to form ordered structures. The polymer with the lowest content of hard segments (PCL-30) was characterized by the highest degree of ordering but only within the domains of soft segments.

#### 3.3.2. TGA

The thermal stability of the obtained SPTURs was determined by means of thermogravimetric analysis carried out under an inert gas atmosphere (helium) and in air.

As can be seen from the data shown in Table 4, the decomposition temperatures (*T*_5_ and *T*_10_) for the helium were slightly lower than for the air conditions, while for the *T*_50_ the differences were more pronounced. When analyzing the *T*_5_ and *T*_10_ values in air, it can be seen that the stability of the polymers decreases with the increase of HS content. In the case of *T*_50_, the opposite tendency was apparent. The clear dependence of the decrease in the thermal stability in helium of polymers on the content of HS can be observed when based on the value of *T*_10_. The greater stability of the obtained polymers in air can be explained by the oxidation processes that stabilize hard segments, therefore the polymer decomposition process in air takes place at a higher temperature than in the case of an inert gas atmosphere [23].

**Figure 7:**
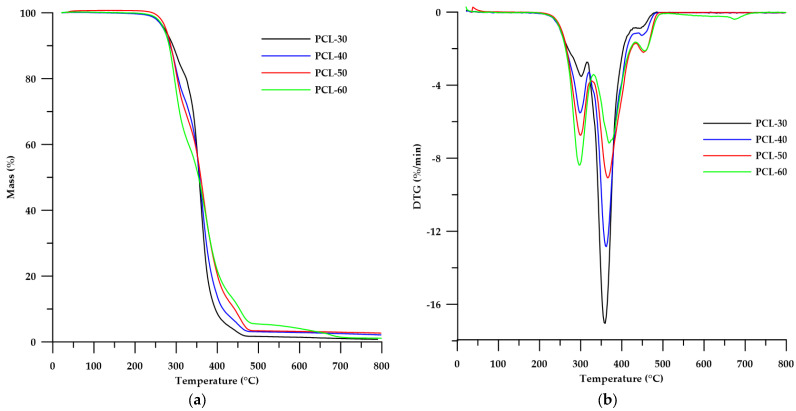
TG (**a**) and DTG (**b**) curves of the obtained polymers (in helium).

**Figure 8:**
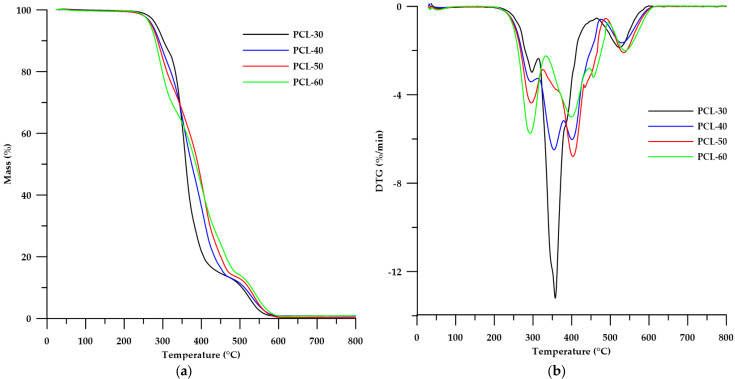
TG (**a**) and DTG (**b**) curves of the obtained polymers (in air).

Taking into account the shapes of the DTG curves (Figure 7b and Figure 8b), we can conclude that the decomposition process of the obtained polymers took place in several stages. The analysis of TG carried out in helium showed that the decomposition of the polymers occurred in three steps (three peaks on the DTG curve), while in the air atmosphere more peaks were visible on the DTG curve. The first peaks (in both helium and air) (*T_max_* with maximas in the range 290–301 °C) increased their intensities with increasing HS content in the polymer, which can be attributed to the distribution of thiourethane bonds of the hard segment [19]. Another peak (decreasing in intensity with increasing HS content in the polymer) in the range of 351–366 °C may be responsible for the degradation of urethane bonds contained in HS and for the decomposition of PCL [39]. Peaks with a *T_max_* of around 405 °C (visible in the DTG curves in the air atmosphere) may be responsible for the distribution of ether bonds contained in the structure of PCL. In turn, the last peaks (increasing in intensity as the HS content of the polymer increases) in both polymer series can be attributed to the degradation of the aromatic fragments derived from the chain extender used. In addition, the presence of broad, low-intensity peaks at about 550–600 °C may account for the oxidation of solid products formed in the initial stages.

#### 3.3.3. DMTA

**Figure 9:**
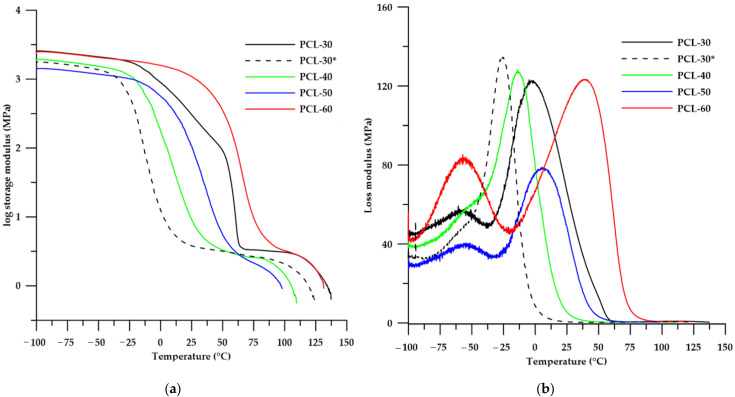

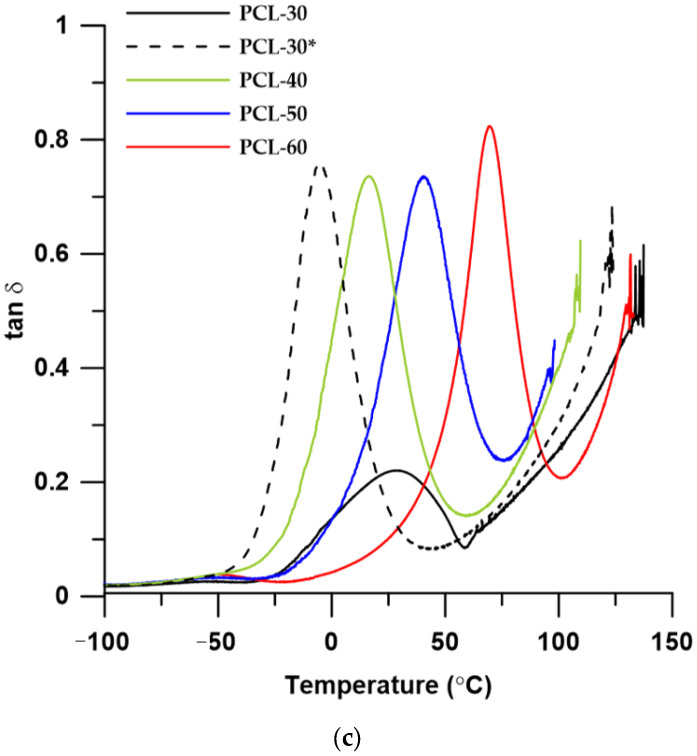
Temperature dependence of storage modulus (**a**), loss modulus (**b**) and tan δ (**c**) of synthesized SPTURs.

To investigate the influence of the soft segments rather than the viscoelastic properties of SPTURs, dynamic mechanical thermal analysis was performed. Changes of storage modulus (*E*′), loss modulus (*E*″) and mechanical loss factor (tan δ) with temperature are shown in Figure 9, while DMTA data are summarized in Table 5.

Due to the fact that the PCL-30 sample shows partial ordering resulting from crystallization of the soft segment, an analysis of the sample being in amorphous form was additionally performed. For this purpose, the compressed sample section was heated in an oven at a temperature above the melting point of the soft segment (i.e., at 60 °C) until the white opacity disappeared completely. Then, for the transparent sample, the test was immediately performed. The results obtained for this sample are shown as dashed lines on the graphs and the sample is coded PCL-30*.

On the basis of the results obtained it can be stated that with an increase in the content of HS in the polymers their glass transition temperature (determined in three different ways: as δ_max_, *E*′_onset_ and *E*″_max_) and the values of storage modulus increased.

Analyzing the shape of the tan δ curve (see Figure 9c) for the PCL-30 sample, it can be seen that the peak with the maximum before annealing has a low intensity (tan δ_max_ of 0.155) and is very broad. This confirms the results obtained from the DSC analysis that this sample shows the highest up-regulation of hard and soft domains. Only the annealing of the sample results in an increase in the entropy of ordering, which is evident by a decrease in peak width (decrease in FWHM value). Similar conclusions were presented in another work [40]. The mixing of the amorphous and crystalline phases in this sample by annealing also caused a decrease in the glass transition temperature and its damping properties (increase in tan δ_max_ value).

Considering the samples in the disordered phase, the tan δ temperature dependence curves (see Figure 9c) show distinct peaks with maxima from −5.47 to 69.15 °C. These correspond to principal relaxations associated with the glass transition in the polymer. Additionally, for the polymers with 50 and 60% wt. HS content at the temperature around −50 °C low intensity peaks are visible, which correspond to local chain movements within the polar thiourethane and urethane groups [20]. These effects are more noticeable on the temperature curve of the loss modulus (see Figure 9b). Clear differences in viscoelastic properties of the polymers obtained can also be observed on the curves of storage modulus vs. temperature (Figure 9a). Analyzing the shape of the curves obtained it can be seen that the PCL-30 polymer after annealing was characterized by the widest range of plateaus. In this range, the polymers exhibit rubber-like properties, and this state is referred to as rubber-elastic. At higher temperatures, further softening of the polymer occurs until a softening temperature is reached, above which polymeric materials can be formed using conventional processing methods. A detailed characterization of the different physical states of polymers during heating was presented in a previous paper [20].

### 3.4. Mechanical Properties

The hardness, tensile strength, elongation at break, modulus of elasticity and lap shear strength are listed in Table 6.

Analyzing the results of the hardness of the polymers obtained it can be stated that the hardness of the polymer increased with an increase in HS content. The exception was the PCL-30 polymer, which had a partially ordered structure resulting from the tendency of the poly(ε-caprolatone) soft segment to self-crystallize (see DSC analysis). The presence of the crystalline phase in the polymer did not influence its other strength parameters.

As shown in Table 6, as the HS content increased, the tensile strength, modulus of elasticity and adhesion to copper increased, while the elongation at break decreased. The increase in adhesion properties of the obtained polymers is largely due to the increase in the presence of sulfur atoms in the polymer [20,41].

In general, the mechanical properties of the obtained polymers are determined by their chemical and phase structure.

### 3.5. Optical Properties

#### 3.5.1. Refractive Index and Transparency

The obtained polymers had refractive indexes values ranging from 1.5325 to 1.5575 (see Table 7), and these values increased with increasing HS content in the polymer. This relationship is in agreement with previous results, which confirm that the refractive index value depends on the presence and amount of sulfur atoms in the polymer: the more sulfur atoms, the higher the polymer’s refractive index. The refractive index values of the described polymers were higher than similar polymers based on other (polyether) soft segments [19].

In contrast to the refractive indexes, the transparency of the obtained polymers decreased with increasing HS content in the polymer. The exception was the polymer PCL-30, which was opaque (see Figure 1c). The polymer with the highest transparency was PCL-40. Comparing the appearance of the obtained polymers (see Figure 1c), it can be observed that the more sulfur atoms in the polymer, the more yellow the polymer becomes. To further characterize the colour-dependence of the HS content in the polymer, a colour test according to ASTM E308 was performed.

#### 3.5.2. Colour

**Figure 10:**
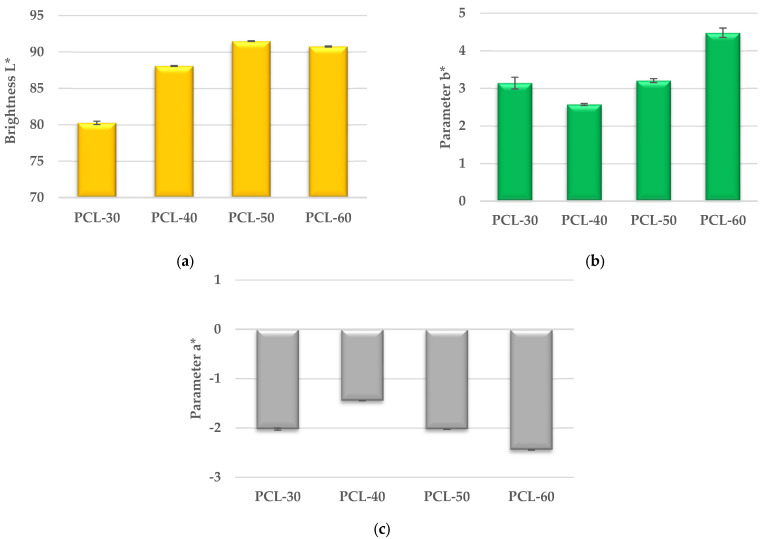
Brightness L* (**a**), Parameter b* (**b**) and Parameter a* (**c**) of synthesized SPTURs.

The results of the color tests of the obtained polymers are shown in Figure 10.

As can be seen from Figure 10a, the brightness L* of the polymers in the series increases (except for the PCL-60 polymer), which indicates that the proportion of white colour in the series increases. On the other hand, the increase in parameter b* in the series (except for sample PCL-30) demonstrates that the proportion of yellow colour also increases, which can be seen by observing the appearance of the samples in Figure 1c. The increase in parameter b* in the series can be related to the increase in the number of sulfur atoms in the polymer, i.e., the more sulfur, the more yellow the polymer. A similar relationship was observed for the parameter a*, the increase of which indicates a shift in the colour of the obtained polymer towards the green colour.

## 4. Conclusions

The high-molar-mass SPTURs with 30–60 wt% of hard segment content were synthesized from nonconventional chain extenders, i.e., (methanediyldibenzene-4,1-diyl)dimethanethiol (dithiol), HMDI and aliphatic poly(ε-caprolactone)diol of ${\bar{M}}_{n}$ = 2000 g/mol via a one-step melt polyaddition. On the basis of the experiment carried out, it may be concluded that the SPTURs were characterized by very good tensile strength (up to 43.26 MPa), hardness up to 96.25/59.00 Sh A/D and an adhesion to copper of 14.66 MPa. The SPTURs showed a relatively good thermal stability. Their *T*_5_s were contained within the range of 271–283 °C (in air) and 270–275 °C (in helium). The SPTURs decomposed in three (in helium) or four/five (in air) stages.

A DSC study showed that the resulting SPTURs had a *T_g_*s in the range of −39–40 °C, and these values depended on the amount of HS in the polymer. Based on the results of DSC and DMTA analysis, it can be concluded that, generally, the polymers at room temperature showed the properties characteristic of elastomers, and with the increase in the amount of HS in the polymer, their microphase separation decreased. The surface properties of the obtained polymers shows that all obtained SPTURs were hydrophilic (CAs for water between 64.07° and 73.12°) with calculated SFE up to 46.69 mN/m.

The best potential candidate among all the tested samples for use as a biomedical material is the PCL-50 polymer. The performed analyzes showed that it is characterized by the best hydrophobicity, high molecular weight and, at the same time, favorable strength properties. However, long-term in vitro and in vivo studies should be performed to further characterize and propose a specific use as biomedical material.

## Figures and Tables

**Table 1:** Designations of the SPTURs.

SPTUR	Amount of Dithiol (mol%)	Amount of Soft Segment (mol%)	^1^ Hard-Segment Content (wt%)
PCL-30	53	47	29.87
PCL-40	67	33	39.83
PCL-50	77	23	50.15
PCL-60	85	15	59.88

^1^ The hard-segment content (wt%) is calculated by using the expression $\frac{W_{dithiol}+W_{HMDI}}{W_{dithiol}+W_{HMDI}+W_{SS}}$, where *W_dithiol_*, *W_HMDI_* and *W_SS_* are the mass of dithiol, HMDI and soft segment, respectively.

**Table 2:** *η*_red_ values and GPC data of the SPTURs.

SPTUR	*η*_red_ (dL/g)	$\bar{M_{n}}(\mathrm{Da})$	$\bar{M_{w}}(\mathrm{Da})$	*Ð_M_*
PCL-30	2.43	74,100	96,000	1.30
PCL-40	2.25	31,800	57,200	1.80
PCL-50	2.19	60,200	95,000	1.58
PCL-60	1.81	84,300	130,000	1.54

**Table 3:** DSC data of the synthesized SPTURs and SS.

SPTUR	*T_g_* [°C]		*T_m_* [°C]		Δ*H* [J/g]	
	I ^a^	II ^b^	I ^a^	II ^b^	I ^a^	II ^b^
PCL-30	-	−39	54; 154	-	50; 0.9	-
PCL-40	−30	−35	49; 169; 180	-	2.1; 0.5; 0.5	-
PCL-50	2	−3	162; 192	-	0.3; 0.1	-
PCL-60	42	13	53; 151; 192	-	2.3; 0.5; 0.4	-
PCL	−63	−67	17; 53	46; 51	2.6; 112.6	92.3

^a, b^ first and second heating scan, respectively.

**Table 4:** TG data of the synthesized SPTURs.

SPTUR	*T*_5_^1^ (°C)		*T*_10_^2^ (°C)		*T*_50_^3^ (°C)		*T_max_*^4^ (°C)	
	Air	Helium	Air	Helium	Air	Helium	Air	Helium
PCL-30	283	272	302	292	360	356	298; 356; 389; 524	301; 359; 443
PCL-40	276	270	291	287	374	357	295; 351; 405; 447; 529	299; 362; 448
PCL-50	274	275	288	286	393	360	296; 351; 406; 452; 534	300; 366; 452
PCL-60	271	272	283	280	386	355	293; 404; 452; 467; 538	290; 365; 454

^1,2,3^ The temperatures of 5, 10 and 50% mass loss, respectively; ^4^ The temperatures of maximum rate of mass loss.

**Table 5:** DMTA results of the SPTURs.

SPTUR	*E*′_onset_ (°C)	*E*′_20_ (MPa)	*E*″_max_ (°C)	*E*″_max_ (MPa)	tan δ_max_ (°C)	tan δ_max_	FWHM (°C)
PCL-30	−10.25	390	−3.78	142.4	27.31	0.155	51.00
PCL-30*	−26.79	4.15	−25.72	133.2	−5.47	0.691	28.84
PCL-40	−16.57	15.05	−13.85	126.5	16.62	0.635	33.73
PCL-50	7.16	171	5.75	78.08	40.06	0.569	31.44
PCL-60	46.45	1211	39.40	123.0	69.15	0.669	23.32

**Table 6:** Hardness and mechanical properties of SPTURs.

SPTUR	Hardness (Sh)		Tensile Strength (MPa)	Elongation at Break (%)	Modulus of Elasticity (MPa)	Lap Shear Strength (MPa)
	A	D				
PCL-30	90.75 ± 1.50	35.00 ± 0.82	30.04 ± 0.85	500 ± 0	0.61 ± 0.02	1.21 ± 0.18
PCL-40	69.25 ± 0.96	21.75 ± 0.96	30.79 ± 0.63	467 ± 14.4	1.30 ± 0.07	5.50 ± 0.56
PCL-50	71.50 ± 0.58	26.50 ± 1.29	39.41 ± 1.78	341 ± 14.4	2.47 ± 0.18	11.80 ± 0.89
PCL-60	96.25 ± 0.96	59.00 ± 0.82	43.26 ± 0.58	200 ± 0	84.81 ± 5.53	14.66 ± 0.75

**Table 7:** Refractive index and transmittance of SPTURs.

SPTUR	Refractive Index	Transmittance (%)	
		*T*_500_ ^1^	*T*_800_ ^2^
PCL-30	- ^3^	1.10 ± 0.002	3.26 ± 0.003
PCL-40	1.5325 ± 0.002	73.79 ± 0.012	82.09 ± 0.005
PCL-50	1.5455 ± 0.003	72.54 ± 0.007	78.47 ± 0.004
PCL-60	1.5575 ± 0.002	70.19 ± 0.009	78.31 ± 0.008

^1,2^ transmittance at 500 and 800 nm, respectively, ^3^ opaque.

## Data Availability

The data presented in this study are available on request from the corresponding author.
